# An ethno-botanical study of medicinal plants used for the management of respiratory tract disorders in northern parts of Palestine

**DOI:** 10.1186/s12906-023-04176-5

**Published:** 2023-10-27

**Authors:** Nuha Shawarb, Manal Badrasawi, Hassan Abu Qaoud, Fatima Hussein

**Affiliations:** 1 Department of Chemistry, Faculty of Science, An-Najah National University Nablus, P.O. Box 7, Nablus, State of Palestine; 2 Nutrition and Food Technology, Faculty of Agriculture and Veterinary Medicine, An-Najah National University Tulkarem, Nablus, State of Palestine; 3 Department of Plant Production and Protection, Faculty of Agriculture and Veterinary Medicine, An-Najah National University Tulkarem, Nablus, State of Palestine; 4 Department of Pharmacy, Faculty of Medicine and Health Sciences, An-Najah National University Nablus, Nablus, State of Palestine

**Keywords:** Ethno botanical, Herbal plant, Decoction, Respiratory disorders

## Abstract

**Background:**

The medicinal application of natural plant remedies is well established. These medicinal plants are still in use within the Palestinian community to treat several illnesses. This research is intended to study the use of natural plants to treat different types of respiratory tract disorders.

**Method:**

This ethno botanical study focused on the medicinal plants that are used to treat respiratory diseases in the northern part of Palestine; Nablus, Tulkarm, Qalqilia, and Jenin. A questionnaire was distributed to 120 respondents. The data obtained included names of the plants used, the parts used, the diseases for which the products were applied, as well as the method of preparation. To evaluate results, percentages (%), Fic (factor of informant consensus), and FL (fidelity-level) were calculated.

**Results:**

A total of 120 participants were selected for the final analysis. The highest percentage of herbal use was reported for flu 85.8% (103 participants) followed by cough 83.3%, while the lowest percentage of users was for bronchitis with 54.1%. The study showed that 31 plant species of 19 families were used for respiratory disorders treatment. Six species were from the *Lamiaceae* family, three species from the *Apiaceae* family, two species from *Amaryllidaceae, Fabaceae, Myrtaceae, Rutaceae* and *Zingiberaceae*, and one plant species for each of the rest of families. Leaves and fruits were the most commonly used parts of plants. Decoction was the method of preparation and was taken as a hot drink. Chamomile, mint, sage, lemon, and ginger were in the recipes for the five respiratory diseases.

**Conclusion:**

In Palestine, patients with respiratory diseases rely heavily on the use of herbal remedies. Leaves and fruits were the most commonly used plat elements. Age and marriage were significantly associated with the use of botanical remedies. Whereas there was no significant association between the source of information about medicinal plants and the location where medicinal plants were purchased. It is vital to conduct comprehensive clinical investigations and pharmacological assessments of these herbal remedies, in order to identify their efficacy, safety, and toxicity levels.

## Background

Natural plant extracts play an important role in the field of research and development of new drugs. Recent studies have recorded the success of using plants in the treatment or prevention of a wide variety of diseases including cardiovascular diseases [1, 2], atherosclerosis [3, 4], diabetes [5, 6], and cancer [7, 8]. Additionally, several types of herbal remedies are used worldwide in chronic disease treatment [9].

Approximately 70–95% of populations in developing countries use herbal medicinal plants for basic healthcare [10]. According to the World Health Organization (WHO), about 65–80% of the world’s population depends mainly on herbal plants to cure several illnesses, which may be related to a lack of access to modern medical facilities or due to the fact that herbal plants are often considered a safe source for health promotion [11]. It has been claimed that the use of herbal treatments is quite widespread in Asian nations: where 52.5% of visits to outpatient clinics in Thailand reported the use of herbal plant treatment, compared to 53.2% in Myanmar, and 43.5% in Vietnam [12].

Furthermore, several studies have explored the factors that are associated with the use of herbal medicine in several communities for different indications [13–15]. Rashrah et al. (2017) reported that using herbal supplements was positively associated with older age (above 70), higher educational level, and having comorbidities [14], while in a study conducted among German adults, using herbal medicine was associated with dissatisfaction of conventional treatment, past good experiences, positive aspects associated with herbal medicine, as well as family traditions [15]. In Ethiopia, using herbal medicine was associated with rural residency areas, illiteracy, and average income [13], while gender, race, educational level, family income and having comorbidities were all associated with herbal medicine use by American adults [16].

Palestine, as a region with great biological diversity, has a wide variability in the traditional herbs that are used in herbal medicine [17]. More than 2600 plant species cover the hills and mountains of Palestine, of which more than 700 are known to be used as medicinal herbal plants or as botanical pesticides [18]. In one study, (Ali- Shtayeh et al. 2000) reported that at least 63 reliable plant species are still in use for the treatment of skin, urinary system, gastric system, prostate disease, cancer and other diseases [19]. Other ethno-pharmacological studies have been conducted among Palestinians to explore their uses of herbal medicine to address different diseases, i.e. gastrointestinal tract [20], urological disorders [21], hair and scalp diseases [22] and for cancer [17].

In the context of a systematic review, it is observed that the utilization of complementary alternative medicine is prevalent. Among the various practices falling under complementary and alternative medicine (CAM), herbal medicines, “Transcendental Meditation”, yoga, acupuncture, and other traditional medicinal approaches are commonly employed in the management of respiratory tract ailments. [23].

With the emphasis on respiratory disorders, herbal remedies are frequently used as a treatment in many parts of the world. For the past 20 years, it has been acknowledged that plants are the main therapeutic sources that are used in some developing nations to treat infectious disorders, particularly upper respiratory tract infections [23–25]. Approximately 13,000 plants have been evaluated in the last 5 years [24]. Herbal medicinal plants regained their popularity for the treatment of asthma, with their efficacy and safety aspects being supported by controlled clinical studies [25]. One study that was conducted in the Eastern Ghats of Andhra Pradesh in India, have identified 84 plants that could offer some active ingredients for the aforementioned respiratory problems [26]. The most common respiratory system diseases are: asthma, allergy, bronchitis, common cold, cough and whooping cough [26], therefore, four of these disorders: asthma, bronchitis, common cold and cough were selected for the evaluation in this study. This study aims to document and evaluate the traditional uses of herbal medicinal plants for various respiratory disorders in Palestine and to evaluate the efficacy of plant species based on a review of the literature.

## Methodology and study instruments

This cross-sectional study was designed to determine the percentage of medical plant users for selected respiratory diseases among adults in the northern part of Palestine. The study was conducted from August to November 2018.

A convenient sample procedure was used to recruit 120 participants using Cochran’s (1963) equation for prevalence studies. The prevalence of medicinal plant use among Palestinians as conducted by Shawahna and colleagues (2017) [27], they found the percentage of medicinal plants user was 54.5%. Sample size = n, where n = (Z α/2 )^2^ p (1 – p)/ Δ2, Δ Assumed to be 10%. Considering 10% drop out the required sample size was 115 participants. Accordingly, the number was rounded to 120 participants. The only inclusion criteria was to be a Palestinian adult residing in the northern part of Palestine; (Jenin, Tulkarm, Nablus and Qalqilia) and consent to participate in the study by filling out a paper-based questionnaire. Participants with missing data were excluded from the final analysis.

### Questionnaire development and validation

The questionnaire was created in accordance with an extensive literature review regarding herbal plants use in respiratory tract diseases from different communities. Furthermore, studies that reported medicinal plants use among Palestinians were also reviewed. The questionnaire of the current study consisted of three sections, the first of which contained socio-demographic information such as age, place of residence, marital status, educational level, employment status, work status and economic status. The second part consisted of two questions related to (1) participants’ regular sources of information regarding the usage of medicinal plants, including whether they consult doctors, pharmacists, Attarine (traditional herbs and spices stores) or they refer to books or social media, (2) participant’s source of purchasing or getting medicinal plants; from pharmacies, Attarine, wild fields or other. The third section consisted of 5 groups of questions regarding the five respiratory disorders. Each of the five questions asked about a single respiratory disease included information on the specific medicinal plants used to treat it, their name, the part of the plant they came from, how they were consumed—whether as a hot beverage, food, topical treatment, or an inhalation—and how long they had been used.

The content validity was evaluated through sending the questionnaire to three experts in the field. Few items were amended based on the experts’ comments and suggestions. The reliability test was done using Chronbach alpha test for the third section (medicinal plant use); the reliability was 0.67, indicated acceptable reliability.

### Statistical analysis

The Statistical package for the social Sciences SPSS, version 21 was used to analyze the collected data. Numbers and percentages described the categorical data; this quantitative method was used to demonstrate the relative importance of species known locally. The following calculation was used to compute the proportion of plant use:

$$\% \,{\rm{plant}}\,{\rm{used}}\,{\rm{ = }}\,\frac{{\sum {\rm{U}} }}{{\rm{N}}}\, \times \,{\rm{100}}\%$$

where U is the total number of cited species; N is the total number of informants.

Chi Square test was employed to examine the association between the categorical variables (users and non- users) and the nominal levels, with significance level 0.05.

The Informants ‘Consensus Factor, or Fic, was used to determine the degree of homogeneity between the data supplied by various informants [28].

Fic = Nur - Nt / (Nur − 1), where Nur is the number of use reports from informants for a particular plant-usage category and Nt is the number of taxa or species that are used for a particular plant usage category for all informants. The values range from 0 to 1, with 1 indicating the highest level of informant consent. For example, if informants employ only a few species, a high degree of unanimity is obtained, and the medical tradition is therefore seen as well defined [29].

The Fidelity Level (FL), which is the percentage of informants who claim to have used a certain plant for the same principal purpose, was computed for the most often reported ailments as follows:

$${\rm{FL}}\,\left( {\rm{\% }} \right)\,{\rm{ = }}\,\left( {{\rm{Np/N}}} \right){\rm{ \times 100}}$$

where Np is the number of informants that claim a use of a plant species to treat a particular disease, and N is the number of informants that use the plants as a medicine to treat any given disease [30] .

## Results

A total of 120 participants completed the questionnaire, participants characteristics are presented in Table 1. Most of the participants were married (68.3%), live in villages (65.8%), have a university degree (64.2%) and currently not employed (58.2%), from different age groups and economic status backgrounds.

**Table 1:** Socio demographic characteristics of the participants presented in n (%)

	Variable	Number	Percentage
Age	18 < 30	37	30.8
	30- <40	16	13.3
	40-<50	31	25.8
	50-<60	28	23.3
	60 and above	8	6.7
Marital status	Single	36	30
	Married	82	68.3
	Other	2	1.7
Living area	City	41	34.2
	Village	79	65.8
Educational level	No formal education	4	3.3
	Primary education	13	10.8
	Secondary education	26	21.7
	University	77	64.2
Work status	Not working	71	59.2
	Office work	39	32.5
	Business	4	3.3
	Worker	6	5.0
Economic status (monthly income ILS)	< 2000	31	26.3
	2000–3000	35	29.7
	3000–5000	42	35.6
	5000 and above	10	8.5

The study revealed that 59.2% of participants usually refer to Attarine as their source of information in regards to medicinal plants, followed by social media platforms (32.5%), while less than 5% refer to health care workers or books.

In regard to the source of purchasing or getting the medicinal plants, 54.2% of participants reported that they purchase the herbs from Attarine, while 21.7% they collect them from the wild field, and only 19.2% obtain it from pharmacies, and as shown in Table 2.

**Table 2:** Information about herbal plants and their sources

Variables		Number	percentage
Source of information about medicinal herbs	Healthcare workers (doctors, pharmacists)	5	4.3
	Attarine	71	59.2
	Social media	39	32.5
	Others (books)	5	4.2
Source of herbs	Pharmacies	23	19.2
	Attarine	65	54.2
	Friends	6	5
	Wild field	26	21.7

The results revealed that flu is the most common symptoms that was treated by herbs 85.8% (103 participants), followed by cough 83.3% (100 participants), sore throat 79.1% (95 participants) and allergy 71.6% (86 participants), while bronchitis was the least common disease that the herbs were used in its management 54.1% (65 participants). These results are supported with the Fic values; Flu with 0.882 and it is the highest followed by cough with 0.848 then sore throat with 0.838 (Table 3).

**Table 3:** Informant consensus factor by respiratory treatment

Ailment category	Number of use reports (N_ur_)	Number of species (N_t_)	FIC*
Flu	103	13	0.882
Allergy	87	16	0.825
Cough	100	16	0.848
Sore throat	94	16	0.838
Bronchitis	65	12	0.828

• Informant Consensus Factor, ***Fic*** = **N**_**ur**_ - **N**_**t**_ /( **N**_**ur**_ -**1**), providing a value between 0 and 1, where ‘‘1’’ indicates the highest rate of informant consensus

**Fig. 1:**
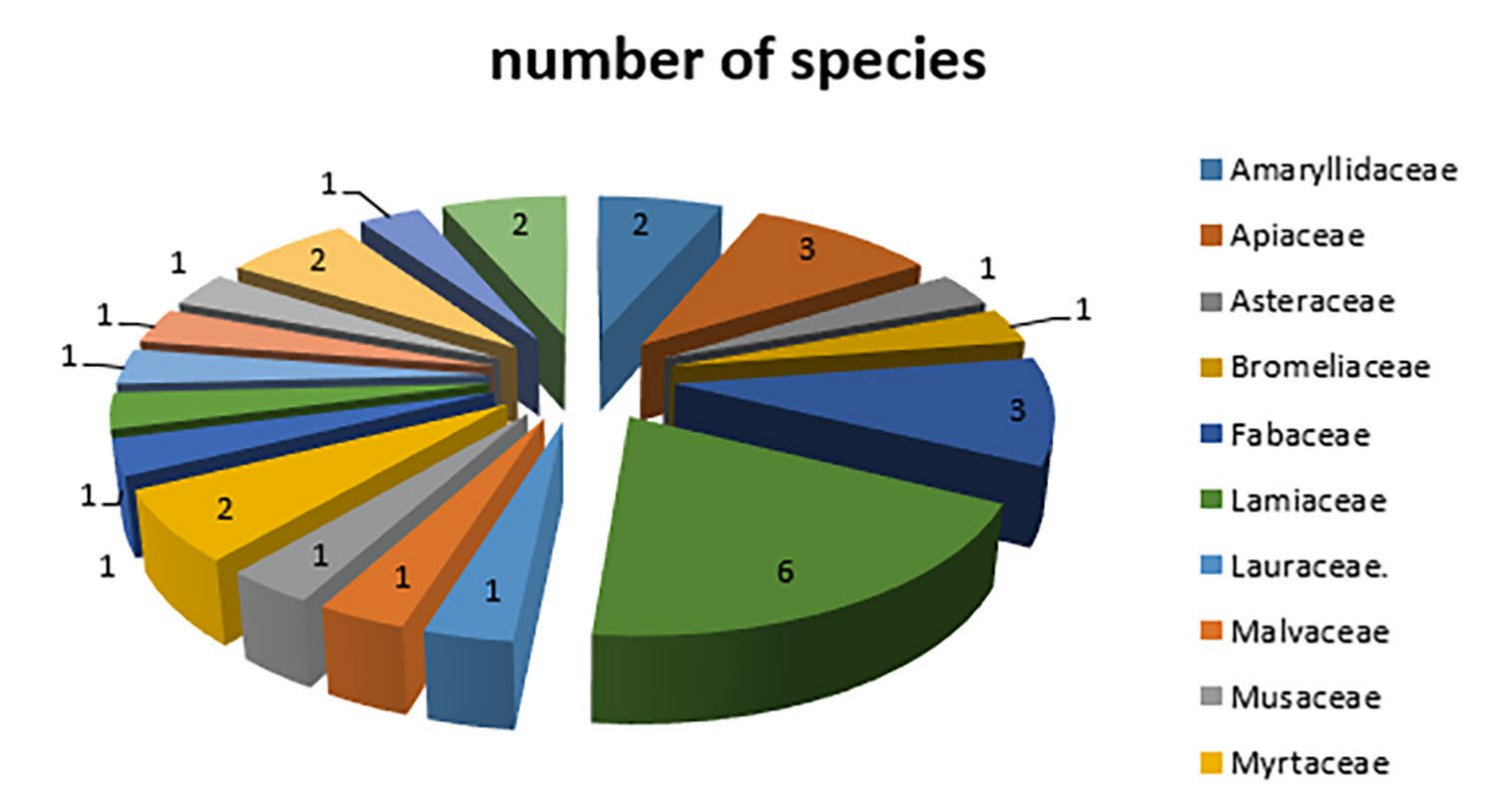
Distribution of the medicinal plants families used by the participants

Figure 1 presents the families and the species for the used medicinal plants by participants, a total of 31 species distributed in 19 families were reported; 6 species are from the *Lamiaceae* family, while 3 species from the *Apiaceae* family, two species are from the *Amaryllidaceae, Fabaceae, Myrtaceae, Rutaceae* and *Zingiberaceae*, while the rest of the families only one species was mentioned by the participants.

Table 4 shows the percentages of users for medicinal plants according to the respiratory diseases. The results showed that the 31 medicinal plants that have been used by the participants for five respiratory disorders. Chamomile, mint, sage, lemon and ginger all were in treatment of all of the five respiratory diseases. Among the least mentioned herbs; parsley and basil. In addition the highest percent of plant used was for lemon with (35%, FL = 62.7) and chamomile with (20.8%, FL = 32) for treating flu, thyme with (34.2%, FL = 62.12) and Guava with (16.7%, FL = 55.6) for cough, ginger with (23.3%, FL = 45.16) for sore throat and finally for bronchitis thyme was used with (15.8%, FL = 28.8), all results of FL values was shown in (Table 5).

**Table 4:** The herbal families and herb species used according to diseases presented in users number and percentages

Family Latin name	Species name Latin	English name	Used parts	Respiratory diseases				
				Flu n(%)	Allergy n(%)	Cough n(%)	Sore throat n(%)	Bronchitis n(%)
*Amaryllidaceae*	*Allium cepa*	Onion	Fruits	-	-	1 (0.8)	1 (0.8)	-
	*Allium sativum*	Garlic	Fruits	-	1 (0.8)	1 (0.8)	5 (4.2)	-
*Apiaceae*	*Pimpinella anisum*	Anise seeds	Seeds	6 (5)	-	1 (0.8)	3 (2.5)	2 (1.7)
	*Petroselinum crispum*	Parsley	Seeds	-	1 (0.8)	-	-	-
	*Cuminum cyminum*	Cumin	Seeds	-	1 (0.8)	-	2 (1.7)	-
*Asteraceae*	*Matricaria chamomilla*	Chamomile	Flowers	25 (20.8)	15 (12.5)	10 (8.3)	12 (10)	16(13.3)
*Bromeliaceae*	*Ananas comosus*	Pinapple	Fruits	-	1 (0.8)	-	-	-
*Fabaceae*	*Trigonella foenum-graecum*	Fenugreek	Seeds	-	1 (0.8)	-	-	-
	*Ceratonia siliqua*	Kharoub	Fruits	-	-	1 (0.8)	-	-
	*Glycyrrhiza glabra*	Licorice root	Stems	-	-	-	4 (3.3)	-
*Lamiaceae*	*Salvia rosmarinus*	Rosemary	Leaves	1 (0.8)	-	-	1 (0.8)	-
	*Ocimum basilicum*	Basil	Leaves	1 (0.8)	-	-	-	-
	*Thymus vulgaris*	Thyme	Leaves	6 (5)	-	41 (34.2)	-	19 (15.8)
	*Salvia officinalis*	Sage	Leaves	2 (1.7)	11 (9.2)	3 (2.5)	4 (3.3)	1 (0.8)
	*Mentha piperita*	Mint	Leaves	4 (3.3)	8 (6.7)	3 (2.5)	2 (1.7)	2 (1.7)
	*Origanum majorana*	Marjoram	Leaves	-	1 (0.8)	-	-	-
*Lauraceae.*	*Cinnamomum cassia*	Cinammon	Stem	-	-	1 (0.8)	2 (1.7)	1 (0.8)
*Malvaceae*	*Malva sylvestris*	Malva	Leaves	-	-	1 (0.8)	-	-
*Musaceae*	*Musa acuminata*	Banana	Fruits	-	-	1 (0.8)	-	-
*Myrtaceae*	*Psidium guajava*	Guava	Leaves	2 (1.7)	4 (3.3)	20 (16.7)	-	10 (8.3)
	*Syzygium aromaticum*	Clove buds	Flower	-	4 (3.3)	2 (1.7)	-	-
*Oleaceae*	*Olea europaea*	Olives leaves	Oil	-	2 (1.7)	2 (1.7)	1 (0.8)	4 (3.3)
*Pedaliaceae*	*Sesamum indicum*	Sesame	Seeds	-	-	-	1 (0.8)	-
*Piperaceae*	*Piper cubeba*	Pipper	Seeds	-	2 (1.7)	-	-	-
*Ranunculaceae*	*Nigella sativa*	Black seeds	Seeds	1 (0.8)	-	-	-	1 (0.8)
*Rubiaceae*	*Cinchona officinalis*	Kenya	Leaves	1 (0.8)	-	-	-	-
*Rutaceae*	*Citrus reticulata*	Orange	Fruits	1 (0.8)	-	-	1 (0.8)	-
	*Citrus limon*	Lemon	Fruits	42 (35)	12 (10)	4 (3.3)	8 (6.7)	1 (0.8)
*Theaceae*	*Camellia sinensis*	Tea	Leaves	-	3 (2.5)	-	2 (1.7)	-
*Zingiberaceae*	*Zingiber officinale*	Ginger	Roots	12 (10)	9 (7.5)	8 (6.7)	28 (23.3)	5 (4.2)
	*Curcuma longa*	Tumeric	Roots	-	-	-	-	2 (1.7)

**Table 5:** Plants used as home remedies for treatment of respiratory diseases in Palestine

Species name Latin	English name	Respiratory diseases				
		Flu n(%)	Allergy n(%)	Cough n(%)	Sore throat n(%)	Bronchitis n(%)
*Allium cepa* *FL*	Onion	-	-	1 (0.8) 50	1 (0.8) 50	-
*Allium sativum* *FL*	Garlic	-	1 (0.8) 14.29	1 (0.8) 14.29	5 (4.2) 71.43	-
*Pimpinella anisum* *FL*	Anise seeds	6 (5) 50	-	1 (0.8) 8.33	3 (2.5) 25	2 (1.7) 16.67
*Petroselinum crispum* *FL*	Parsley	-	1 (0.8) 100	-	-	-
*Cuminum cyminum* *FL*	Cumin	-	1 (0.8) 33.33	-	2 (1.7) 66.67	-
*Matricaria chamomilla* *FL*	Chamomile	25 (20.8) 32.10	15 (12.5) 23.10	10 (8.3) 12.82	12 (10) 15.39	16(13.3) 20.51
*Ananas comosus* *FL*	Pinapple	-	1 (0.8) 100	-	-	-
*Trigonella foenum-graecum* *FL*	fenugreek	-	1 (0.8) 100	-	-	-
*Ceratonia silique* *FL*	Kharoub	-	-	1 (0.8) 100	-	-
*Glycyrrhiza glabra* *FL*	Licorice root	-	-	-	4 (3.3) 100	-
*Salvia Rosmarinus* *FL*	Rosemary	1 (0.8) 50	-	-	1 (0.8) 50	-
*Ocimum basilicum* *FL*	Basil	1 (0.8) 100	-	-	-	-
*Thymus vulgaris* *FL*	Thyme	6 (5) 9.09	-	41 (34.2) 62.12	-	19 (15.8) 28.78
*Salvia officinalis* *FL*	Sage	2 (1.7) 9.50	11 (9.2) 50	3 (2.5) 13.64	4 (3.3) 15.38	1 (0.8) 4.54
*Mentha piperita* *FL*	Mint	4 (3.3) 21.05	8 (6.7) 42.10	3 (2.5) 15.79	2 (1.7) 10.53	2 (1.7) 10.53
*Origanum majorana* *FL*	Marjoram	-	1 (0.8) 100	-	-	-
*Cinnamomum cassia* *FL*	Cinammon	-	-	1 (0.8) 25	2 (1.7) 50	1 (0.8) 25
*Malva sylvestris* *FL*	Malva	-	-	1 (0.8) 100	-	-
*Musa acuminate* *FL*	Banana	-	-	1 (0.8) 100	-	-
*Psidium guajava* *FL*	Guava	2 (1.7) 5.56	4 (3.3) 11.11	20 (16.7) 55.56	-	10 (8.3) 27.78
*Syzygium aromaticum* *FL*	Clove buds	-	4 (3.3) 66.67	2 (1.7) 33.33	-	-
*Olea europaea* *FL*	Olives leaves	-	2 (1.7) 22.22	2 (1.7) 22.22	1 (0.8) 11.11	4 (3.3) 44.44
*Sesamum indicum* *FL*	Sesame	-	-	-	1 (0.8) 100	-
*Piper cubeba* *FL*	Pipper	-	2 (1.7) 100	-	-	-
*Nigella sativa* *FL*	Black seeds	1 (0.8) 50	-	-	-	1 (0.8) 50
*Cinchona officinalis* *FL*	Kenya	1 (0.8) 100	-	-	-	-
*Citrus reticulate* *FL*	Orange	1 (0.8) 50	-	-	1 (0.8) 50	-
*Citrus limon* *FL*	Lemon	42 (35) 62.69	12 (10) 17.91	4 (3.3) 5.97	8 (6.7) 11.94	1 (0.8) 1.49
*Camellia sinensis* *FL*	Tea	-	3 (2.5) 60	-	2 (1.7) 40	-
*Zingiber officinale* *FL*	Ginger	12 (10) 19.35	9 (7.5) 14.51	8 (6.7) 12.90	28 (23.3) 45.16	5 (4.2) 8.06
*Curcuma longa* *FL*	Tumeric	-	-	-	-	2 (1.7) 100

**Fig. 2:**
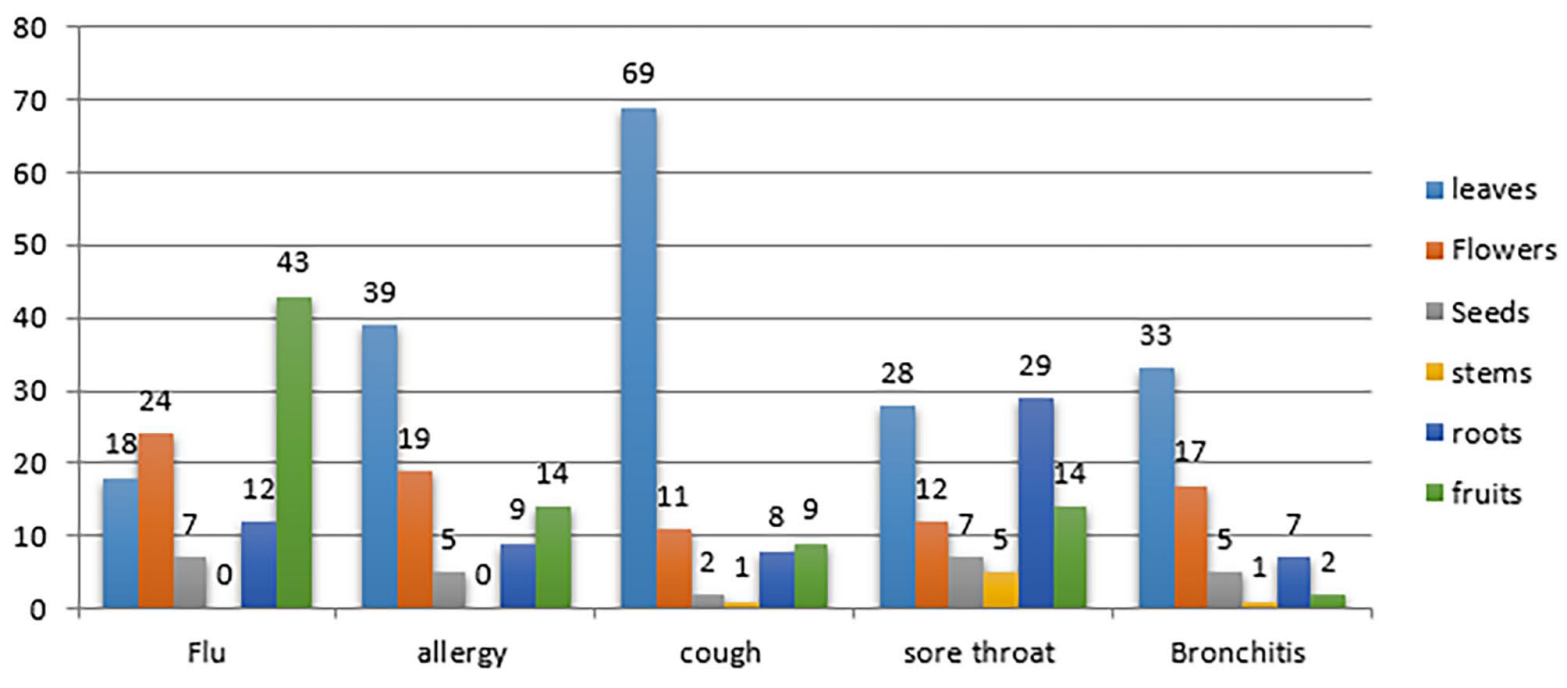
Parts of the plant used according to diseases

Leaves were the most common used part used of the plant for most respiratory disorders, followed by the fruits. Even there are variety of the medicinal plants parts used according to disease as shown in Fig. 2.

**Fig. 3:**
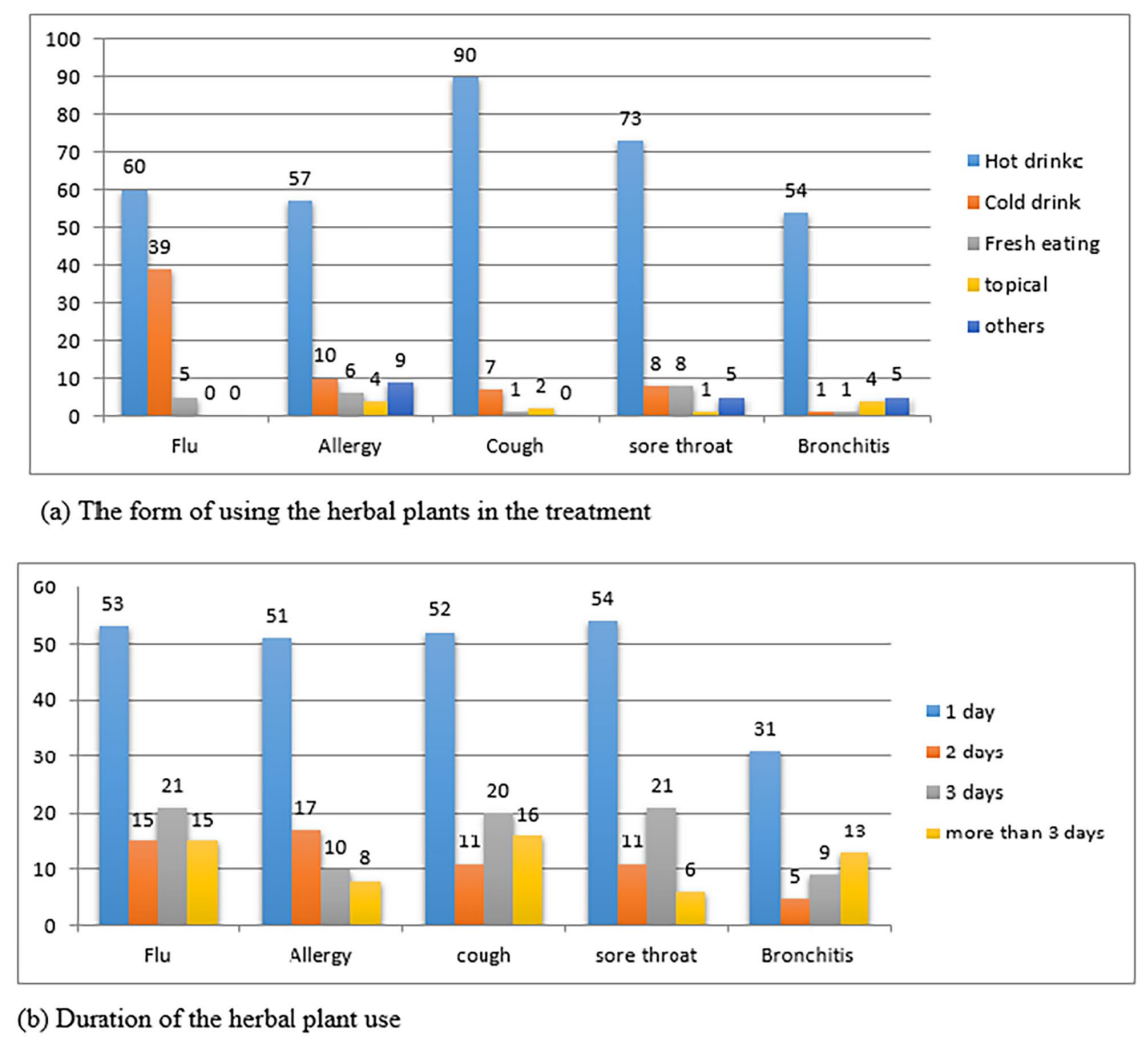
The consumption of herbal plants from and duration of consumption

Figure 3a. Shows that the most common way of consuming medicinal plants was hot beverages, followed by cold drink then fresh eating, while topical administration was the least used approach. In relation to the duration of medicinal plants used, the short duration for only 1 day was the most common period of taking the herbs (as around 50% of the participants reported that they take the herbs for 1 day in most of the diseases, then for three days; around 20% of the participants reported that they use the medicinal plants for three days in all of the respiratory diseases, while for use more than three days the range was from 10 to 15% of the participants reported that they use the medicinal plant for period longer than three days (Fig. 3b).

### Traditional treatment of respiratory disorders using plants extracts

Finally, it was seen that the respiratory system ailments, for which the folk medicinal plants were mostly used, were as follows: (flu, allergy, cough, congestion and bronchitis). Informant consensus of medicinal plant usage resulted in informant consensus factor (Fic) values between 0.825 and 0.882 per respiratory disorders category. The category that had the highest Fic value was flu (0.882) followed by cough (0.848). The lowest was allergy and bronchitis (Table 3).

It could be concluded that the plants with high Fic values will be transferred more and therefore could be utilised much better in treatment of certain illnesses [31]The average Fic value for all respiratory disorder categories was 0.844, indicating a fairly high level of informant consensus compared with similar studies.

### Differences between users and non-users of medicinal plants

Further analysis was done to determine the association between medicinal plants uses in treating the five respiratory diseases and all of the collected socio-demographic variables, using univariate analysis (Chi Square test). The results revealed that being older than 40 years old was significantly associated with higher use of medicinal plants in the treatment of flu and cough as compared to being younger than 40 years old (p < 0.05). While the association was not significant across different age groups for the rest of respiratory diseases; allergy, sore throat and bronchitis. In regard to the marital status, being married was significantly associated with higher use of medicinal plants for flu treatment، compared to being single (p < 0.05).

While the association was not significant between being users or non-users of medicinal plants in any of the respiratory diseases neither with the source of Information about the medicinal plants nor with the place of purchasing the medicinal plants.

## Discussion

This study successfully determined the prevalence of herbal medicinal plants uses for the treatment of the selected five respiratory diseases. This includes the types of medicinal plants used in addition to the parts of the plants used, and the method of use, among a representative sample of Palestinian adults and older adults from different residency areas. To our knowledge, this is the first study in Palestine to explore the use of medicinal plants in respiratory diseases treatments.

This present study showed higher use of medicinal plants among participants aged 40 years and older, which is consistent with the findings from Rashrash, M., J.C. Schommer, and L.M. Brown.et,al (2017) [14], who also found that using herbal medicinal plants is common among middle- and older-aged people. In addition, using medicinal plants was associated with being married, which may also be explained due to the age factor, as single participants are generally younger in age. One study that was conducted by Kelly, J.P., et al. (2005) [32] reported that among the 8470 included participants, there are differences in herbal medicinal plants uses across different age and gender groups [32]. These differences may be due to the assumption that older people believe in traditional medicine more than modern and westernized medicine, mainly in terms of safety and efficacy with fewer side effects [15].

Table 4shows the natural herbal plants used to treat respiratory tract disorders presented in user numbers and percentages, families of which these herbs belongs and part used. According to our findings, as stated in Table 4, there was 31 different plant species distributed in 19 families, the plants most frequently used were members of the *Lamiaceae* family with 6 species followed by the *Apiaceae* family with 3 species, *Lamiaceae* also was first according to a study carried out in Middle Region of Oum Rb [33]. Leaves were the part used in the *Lamiaceae* herbal plants, so it has the highest percentage as used part according to Fig. 2 with 69% for treating cough followed by allergy with 39%,this could be explained as leaves are the photosynthetic organs containing the effective bioactive ingredients. [34], followed by fruits with 43% for treating flu. It has also been reported in different studies that Americans are commonly using Fruit also [35]. Seeds were the part of the plant used in the family *Apiaceae* ranked third, other plant parts used including; flowers, stems and roots.

According to (Fig. 3a): decoction was the method of preparation, it means heating the herbs all or a specific part of it in water to boiling for few minutes and taken orally as a hot drink. Sometimes a mixture of more than one plant species of a family or more may be used to insure a better efficacy. Previous studies reported that decoction and infusion predominates as a method of preparation for many respiratory disorders treatments [36].

In the current study, the most prevalent respiratory tract condition treated with herbal medicinal plants was flu with (103 participants),a common result reported in west Iran were 23 medicinal plants used in Lorestan province for treating cold [37].

followed by cough (100 participants) and sore throat (94 participants) [33], these results were proved by calculating Fic values (Table 3) where it was the highest 0.882 for flu followed by cough with 0.848 and sore throat 0.838. This may be due to the high common spread of these disorders among people in Palestine mainly in winter season, or could be related to the previous observations of the susceptibility of these disorders to herbal medicinal therapy. While bronchitis was, the least common disease that treated by herbs because in case of more serious lung diseases like bronchitis; the population uses less herbal plants, and prefer using medical prescriptions by doctors.

As aforementioned, certain plants were used in the treatment of the selected five respiratory diseases. These were mainly: (1) Chamomile that belongs to the *Asteraceae* family, (2) Mint and sage from the *Lamiaceae* family, (3) lemon that belongs to the *Rutaceae* family and (4) ginger from the *Zingiberaceae* family. Accordingly, these medicinal plants can be considered as an indication of their high healing potential against related diseases. Plants with high percentages and FL values were; lemon with (35%, FL = 62.7) and chamomile with (20.8%, FL = 32) for treating flu, thyme with (34.2%, FL = 62.12) and Guava with (16.7%, FL = 55.6) for cough, ginger with (23.3%, FL = 45.16) for sore throat and finally for bronchitis thyme was used with (15.8%, FL = 28.8). Many other previous studies reported similar results in relation to the efficacy of these plants as home remedies for the treatments of respiratory diseases; Chamomile have been reported for treating upper respiratory tract diseases, in the United States, which also known to be one of the most widely consumed form of tea [38].

This finding was supported by ESSAIH, S., et al. 2023, who reported that among 78 species of vascular plants from 34 families a significant representativeness of the *Asteraceae* (12%) were recorded with 60% uses of the aerial part of the plant as medicinal uses [39].

Mint and sage are native to the Mediterranean region and have been used worldwide as flavoring spices as well as traditional herbal medicine for common respiratory disorders [40, 41], findings of a local study showed that the essential oils isolated from lemon, *M. spicata* leaves and *C. sinensis* flowers enhanced athletic performance and lung function [42], Ginger have been proven to relieve coughing [43], Thyme extract was approved to help patients with chronic obstructive pulmonary disease [44], Leaves of guava were mentioned as a remedy for cough during an ethnobotanical survey in Guerrero, México [45] in Malaysia, [46] and in South Africa [47].

As a result, the bioactive components of these plants need to be investigated, and this will be the focus of more phytochemical research in the near future. In addition, detailed information regarding the gathering and processing of each plant remedy needs to be recorded.

## Conclusion

In Palestine, patients with respiratory tract diseases frequently use herbal medicinal plants. It was found that the local population uses 31 plants from 19 separate families. Most of them grow in the wild, and some are cultivated, from these medicinal plants. (i.e. *mint, sage and thym*). People use these plants by drying, decoction or infusion during all seasons of the year. Chamomial belongs to *Asteraceae* family, mint and sage for *Lamiaceae* family, lemon belongs to *Rutaceae* family and ginger belongs to *Zingiberaceae* family all were agreed to be used in treatment of all of the five respiratory diseases. The most commonly used sections of the plants were leaves and fruits. The quality of plant species fidelity and informant consensus factor values for plants have been developed. The high mean value of Fic (0.844) suggest substantial level of homogeneity among the data provided by different respondents. There was a correlation between becoming older and getting married when using botanical treatments. Whereas there does not appear to be any substantial connection between the source of information about medicinal plants and the region from where medicinal plants are acquired. The findings of this study emphasize the significance of conducting additional research on these herbal medicinal plants, specifically through complete clinical investigations and pharmacological evaluations. These measures are necessary to determine the safety, toxicity, and efficacy of these treatments.

## Data Availability

Data are all contained with the article.
